# Fast Independent Component Analysis Algorithm-Based Functional Magnetic Resonance Imaging in the Diagnosis of Changes in Brain Functional Areas of Cerebral Infarction

**DOI:** 10.1155/2021/5177037

**Published:** 2021-11-28

**Authors:** Naiyi Du, Zhao Zhang, Yao Xiao, Lijie Jiang

**Affiliations:** Imaging Center, The People's Hospital of Hengshui City, Hengshui, Hebei 053000, China

## Abstract

The aim of this study was to analyze the application value of functional magnetic resonance imaging (FMRI) optimized by the fast independent component correlation algorithm (ICA algorithm) in the diagnosis of brain functional areas in patients with lumbar disc herniation (LDH). An optimized fast ICA algorithm was established based on the ICA algorithm. 50 patients with cerebral infarction were selected as the research objects, and 30 healthy people were selected as the control group. The 50 patients from the observation group were examined by fMRI based on Fast ICA algorithm, while the control group was tested by fMRI based on the routine ICA algorithm. The performances of the two algorithms, the analysis results of the two groups of brain functional areas, cerebral blood flow (CBF), resting state functional connectivity (rsFC), behavioral data, and image data correlation of patients were compared. The results showed that the sensitivity, specificity, and accuracy of Fast ICA algorithm were 97.83%, 89.52%, and 96.27%, respectively, which in the experimental group were greatly better than the control group (88.73%, 72.19%, and 89.72%), showing statistically significant differences (*P* < 0.05). The maximum Dice coefficient of FAST ICA algorithm was 0.967, and FAST ICA algorithm was better obviously than the traditional ICA algorithm (*P* < 0.05). The cerebral blood flow of the healthy superior frontal gyrus (SFG) and healthy superior marginal gyrus (SMG) of the observation group with good motor function recovery were 1.02 ± 0.22 and 1.53 ± 0.61, respectively; both indicators showed an increasing trend, and those in the experimental group were much higher in contrast to the control group, showing statistically obvious differences (*P* < 0.05). Besides, the detection results of cerebral blood flow (CBF) in the healthy SFG and healthy SMG were negatively correlated with the results of connection test B. In summary, the fMRI based on the Fast ICA algorithm showed a good diagnostic effect in the changes of brain functional areas in patients with cerebral infarction. The experimental results showed that the cerebral blood flow in the brain area was related to motor or cognitive function. The results of this study provided a reliable reference for the examination and diagnosis of brain functional areas in patients with cerebral infarction.

## 1. Introduction

In recent years, with the continuous improvement of people's living standards, the incidence of heart and brain diseases has also increased year by year, especially the number of patients with cerebral infarction. Cerebrovascular stenosis and occlusion lead to ischemic hypoxia necrosis of brain cells, which results in corresponding brain tissue necrosis, abnormal brain structure, functional and energy metabolism, and clinical dysfunction of motor, sensory, and cognitive functions [1, 2]. The brain is a complex structure consisting of neurons in the cortex and subcortex, which interact to maintain the balance of brain functions. Ischemic infarction of the brain motor pathway may lead to severe dysfunction of the motor system [3, 4]. The plasticity and reorganization ability of the brain after cerebral infarction is a necessary condition for the recovery of patients' neurological function, which is currently a hotspot of neuroscience research. It has important research significance and clinical significance and helps to promote the prognosis and rehabilitation of patients [5, 6].

The human brain is not only plastic in structure, but also has the ability of recombination in pathophysiology. When local cerebral ischemia and hypoxic necrosis occur, local brain structures (such as white matter volume, gray matter volume (GMV), cerebral cortical thickness, and curvature) and brain functions (such as resting state functional connectivity (rsFC), amplitude of low-frequency fluctuation (ALFF), and regional homogeneity (Reho)) can also be restored. The research on changes and reorganization of brain function after cerebral infarction is of practical value in evaluating the prognosis and functional recovery of patients [7]. Nowadays, more instruments are applied to examine the functional areas of the brain such as functional magnetic resonance imaging (fMRI) based on blood oxygenation level dependent (BOLD) [8]. fMRI is divided into two modes, namely, task-state fMRI and resting-state fMRI. Resting-state functional connectivity (rsFC) analysis method is often applied in the overall study of brain networks based on resting-state fMRI [9, 10].

In recent decades, fMRI technology has been widely used in the field of science and technology, and the cognitive network of the brain plays an irreplaceable role in accurately detecting brain function [11]. However, it is more complicated to extract the basic characteristic elements from the mixed signal when using fMRI detection, and there are many interference factors, such as noise signals, task-related signals, and eye movement signals. If the true brain activity signal and other uncorrelated signals such as noise signals are unrelated, the collected statistical MRI signals are independent of each other and the collected time series can be regarded as each independent mixed element [12]. Since the reason why unknown activities activate the brain is difficult to determine, the independent signal source and the mixing matrix are unknown, which belongs to a typical blind source separation problem. The independent component analysis (ICA) method is often employed to solve the blind source separation problem. What is more, ICA is a method of separating components from a large amount of mixed data, which is applied to the fMRI examination of patients with cerebral infarction, which has a significant optimization effect [13].

Based on the above, there are not too many relevant research conclusions in the past. This study firstly constructed an improved independent component analysis algorithm and applied it to fMRI to provide reference for the diagnosis of brain functional area changes in patients with cerebral infarction.

## 2. Materials and Methods

### 2.1. Research Objects and Grouping

In this study, 50 patients with cerebral infarction who admitted to hospital for treatment from July 27, 2019, to July 27, 2020, were selected as the research objects. There were 40 males and 10 females, aged 40–5 years old, with an average age of 55.21 ± 6.33 years old. The studies involving human participants were reviewed and approved by the ethics committee of hospital. The patients/participants provided their written informed consent to participate in this study.

The criteria for inclusion were defined to include patients who suffered from chronic cerebral infarction, with a course of more than 6 months, had cerebral infarctions in the subcortical area (all onset for the first time), had no other lesions or mental diseases in the brain, had no major physical or tumor diseases, and could ensure high cooperation and good communication.

The criteria for exclusion were defined to include patients who suffered from multiple or recurrent cerebral infarction, had the lesion in the brain, had severe white matter demyelination disorder, and suffered from alcohol dependence or mental illness.

In addition, 30 healthy people of the same age and gender who underwent physical examination at the same time were recruited as the control group. The included research objects had brain lesions or structural abnormalities in the examination department, did not have the habit of smoking or drinking, and did not use medicine or drugs.

50 patients from the observation group were tested by fMRI based on the Fast ICA algorithm, and the routine ICA algorithm-based fMRI was used for the detection of the research objects from the control group.

### 2.2. Assessment of the Patients' Cognitive Level

The computer version of Trail Making Test (TMT) Part A (TMT-A) and Part B (TMT-B) was adopted in this study. Connection TMT-B: some numbers and letters would be displayed on the computer screen, such as 1, 2, 3, 4, ..., 13 and A, B, C, D, E, F, G, H, I, J, K, L, and numbers and letters were scattered randomly distributed [14]. When the test was started, the patient was instructed to sit in front of the computer, look up at the screen, and use the left mouse button to quickly click the corresponding numbers and letters in the order of A-2-B-3-C-4-D……11-K-12-L-13 to record the time for the patient to complete the test. The connection TMT-A reflected the speed of the research objects to process information. The shorter the time it took the patient to complete connection TMT-A, the faster the research objects process the information. The connection TMT-B meant the patient's execution ability. The shorter the time for the patient to complete the connection TMT-B, the stronger the execution ability of the research object.

### 2.3. Parameter Basis of fMRI Equipment

The detection equipment applied in this study included the 3.0 T magnetic resonance scanner and the head eight-channel phased array coil scanning. All patients in this study were scanned with three-dimensional (3D)-BRAVO sequence, 3D-pseudo continuous arterial spin labeling (pcASL) sequence, and REST sequence.

### 2.4. Experiment Process

Before the start of the experiment, the researchers should explain the experiment process for the participants and all participants should sign the informed consent forms to ensure full cooperation. The simplified Fugal–Meyer motor function gradient method was adopted to evaluate the motor function and cognitive function of the participants. With the help of computer evaluation, the participants were explained to complete the evaluation and instructed to take the record of score and time seriously. They should be informed of the time and precautions required for this MRI scan before the scan. During the scan, each participant was instructed to lie on the examining bed, straighten the body, and hold the head in place to reduce head movement. Moreover, the participants were instructed to close their eyes and relax, but remained awake, did not think actively, and kept silent. Sagittal 3D-BRAVO sequences, axial static fMRI, and axial 3D-pcASL sequences were used for scan [15].

### 2.5. The Construction Process of Fast ICA Algorithm

#### 2.5.1. ICA Principle

ICA is a new way of analyzing signals evolved from principal component analysis. The ICA algorithm evolved from the “cocktail party problem” [16]. The general model is as follows:

Suppose *K*=*k*_1_, *k*_2_, *k*_3_, ⋯, *k*_*n*_ is *n* observation signals and *S*=*s*_1_, *s*_2_, *s*_3_, ⋯, *s*_*n*_ is *n* source signals, of which the observation signal *K* is developed from the source signal *S*. Assuming that the *l*th observation signal obtained by mixing the *l*th independent component of the source signal *S*, the following equation can be obtained, where *l*=1,2,3,…, *n*:

(1) $$\begin{array}{c} k_{l}=k_{1}s_{1}+k_{2}s_{2}+k_{3}s_{3}+\cdots +k_{l}s_{n}. \end{array}$$

*X*=[*y*_1_,  *y*_2_,  *y*_3_, ⋯, *y*_*n*_] is supposed, so the relationship between the observation signal *K* and the source signal *S* is as follows:

(2) $$\begin{array}{c} K=XS. \end{array}$$

Equation (2) can be regarded as a general mixed model of ICA. In the above equation, *X*=[*y*_1_,  *y*_2_,  *y*_3_, ⋯, *y*_*n*_] is the mixed matrix. Since each source signal *S* is assumed to be independent of each other, a linear transformation matrix can be found isomorphically to transform the observed signal, so that a signal similar to the source signal *K* is obtained as follows:

(3) $$\begin{array}{c} K=\text{US}=\text{UXS}. \end{array}$$

In equation (3), *U* represents the unmixing matrix. The estimation of the mixing matrix *U* is shown in the following equation:

(4) $$\begin{array}{c} X^{\prime }=U^{-1} \end{array}$$

#### 2.5.2. Fast ICA Algorithm

According to the estimated source signal of the observed signal, a new objective function *P*(*a*) can be established, where *a* stands for the extreme value of the objective function [17]. Kurtosis is the most common standard used by ICA, and the equation for defining random variables is as follows:

(5) $$\begin{array}{c} \kappa \left(a\right)=\frac{W\left\{{\left|s\right|}^{4}\right\}-2W^{2}\left\{{\left|s\right|}^{2}\right\}-W{\left\{\left|s^{2}\right|\right\}}^{2}}{W^{2}\left\{{\left|s\right|}^{2}\right\}}. \end{array}$$

In equation (5), *W*{·} means mathematical expectation, and there is *κ*(*ℓa*)=*κ*(*ℓa*), ∀*ℓ* ≠ 0.

In order to simplify the source signal obtained, whitening can be used to make the zero mean value of the observed signal, as shown in the following equation:

(6) $$\begin{array}{c} H_{s}\overset{\text{def}}{=}W\left\{SS^{I}\right\}=O. \end{array}$$

In the case of real numbers, equation (7) is equivalent to the fourth-order accumulation equation:

(7) $$\begin{array}{c} T\left(a\right)=W\left({\left|s\right|}^{4}\right). \end{array}$$

When ‖*a*‖=1, the fixed point of equation (8) can be found through *W*{*skk*^*∗*2^}, and *ℓ* represents a Lagrangian number.

(8) $$\begin{array}{c} W\left\{a^{I}{S|}^{2}SS^{I}\right\}a=\ell a. \end{array}$$

The kurtosis optimization algorithm mainly adopts the time stochastic gradient algorithm. For the case of maximizing the objective function and minimizing the objective function, the value of *a* should be separated first. In the case of real numbers, the Hessian matrix approximation of *T*(*a*) can be considered as follows:

(9) $$\begin{array}{c} W\left\{\left(a^{i}SS^{i}a\right)SS^{i}\right\}\approx W\left\{a^{i}SS^{i}a\right\}W\left\{SS^{i}\right\}=a^{i}a=O. \end{array}$$

Therefore, Fast ICA based on kurtosis can be transformed into the following equations:

(10) $$\begin{array}{c} a^{+}=a-\frac{1}{3}W\left\{S{\left(a^{i}S\right)}^{3}\right\}, \end{array}$$

(11) $$\begin{array}{c} \frac{a^{+}\leftarrow a^{+}}{\left\|a^{+}\right\|}. \end{array}$$

∇*T*(*a*)=4*W*{*S*(*a*^*i*^, *S*)^3^}, so equation (11) can be converted to

(12) $$\begin{array}{c} a^{+}=a-\partial \nabla T\left(a\right). \end{array}$$

The Fast ICA algorithm has many advantages, such as fast convergence speed and simple calculation, so the application of this algorithm is more common.

### 2.6. Image Processing

Before data preprocessing, the images of 10 patients with subcortical infarction lesions located on the right side were selected by MATLAB software and turned to the left side. Besides, the left side was defined as the ill side, and the right side was defined as the healthy side.

#### 2.6.1. Patient Perfusion Imaging Data Processing

The SPM8 software based on the MATLAB platform was used to process the perfusion image, which could be divided into the following steps. First, the cerebral blood flow (CBF) image was obtained from ASL perfusion image. Then, spatial registration was carried out to align the data of different patients into the same standard space, thus solving the problems of brain morphological differences of different patients and inconsistent spatial positions during scanning. The affine transformation matrix of brain tissue and MNI space generated by the segmentation of patients' structural images was applied to register the perfusion images of the participants to the ICBM161 standard brain Atlas, and then the normalization processing was carried out. Finally, in order to reduce the influence of registration deviation, increase the signal-to-noise ratio of the data, and make the data more consistent with the Gaussian distribution, 3D Gaussian kernel convolution operation was employed to carry out spatial smoothing processing on fMRI, and the FWHM value was 9 × 9 × 9 mm³ [18].

#### 2.6.2. Resting-State fMRI Data Processing

The DPARSF software based on MATLAB platform was used to preprocess the resting state fMRI data. The following steps were carried out [19]: (1) Deletion of previous time: the signal was unstable at the beginning of the collection, and the participants had to adapt to the environment, so it was generally necessary to delete the images taken in the first 10 time stages. (2) Section time correction: MRI images were scanned layer by layer, so the acquisition time of each layer was different. Since the time series needed to run, time scale correction was required to ensure that the capture times for all pixels in the volume were theoretically consistent. Slice timing setting: the total number of layers in this study was 32, and the scanning order was 1 through 32. The reference layer usually had the number of layers corresponding to the scanning center (time center) (i.e., 31 layers or 2 layers). (3) Head movement correction: it was adopted to correct the slight head movement between the volume blocks in the scanning process. In this study, 6 motion parameters, namely, translation and rotation in *x*-, *y*-, and *z*-directions, were estimated by rigid body transformation. (4) Spatial standardization: the data of different participants were aligned to the same standard space to solve the problems of morphological differences among different participants and inconsistent spatial positions in the scanning process. After spatial standardization, all participants' anatomies were theoretically the same and voxel-based statistical comparisons could be made within the standard range. (5) Elimination of linear drift: with the extension of time, there would be a linear trend due to the heat generated in the working process of the machine or fatigue caused by long-term scanning work. (6) Band-pass filtering: the low-frequency component of the BOLD signal mainly reflected the spontaneous neural activity of the brain. The BOLD signal in this part fluctuated slightly and was easily masked by other signals. Band-pass filtering could eliminate low-frequency physiological signals (such as breathing and heartbeat) and high-frequency random noise. In this study, low-frequency signals were screened in the frequency range of 0.01–0.08 Hz. (7) Regression interference signal: the linear regression model was applied to remove the interference information in the BOLD signal. (8) Spatial smoothing: in order to reduce the impact of registration deviation, improve the signal-to-noise ratio of the data, and keep the data consistent with the Gaussian distribution, the spatial smoothing adopted 3D Gaussian kernel convolution, and the FWHM value was 9 × 9 × 9 mm³ [20].

#### 2.6.3. Resting Functional Connection

On the MATLAB platform, REST software was used to perform seed point analysis on the preprocessed functional data. According to the statistical results of CBF, the region of interest (ROI) was selected as the brain region with significant difference in CBF between the observation group and the normal control group. The acute Pearson correlation analysis between ROI and other brain voxels was calculated to obtain the correlation map and correlation coefficient *z*. The correlation map was transformed into *Q* map by Fisher-*Z* transformation, making it conform to normal distribution [21]. The conversion equation was expressed as follows:

(13) $$\begin{array}{c} Q=0.5\;\log\frac{1+z}{1-z}. \end{array}$$

### 2.7. Statistical Analysis

After preprocessing, the CBF value of each voxel of the whole brain of all participants was obtained. The GLM model of SPM8 software was adopted in this study; gender, years of education, and age were taken as covariables, and whole brain gray matter template was used as mask to conduct independent sample *t*-test (*α* = 0.05 as the test level). Besides, the difference in CBF between the observation group and the normal control group was compared, and the results were expressed as mean ± standard deviation. The threshold for each voxel was set at *P* < 0.005 (two-sided). When *P* < 0.05, the difference was considered to be statistically substantial. In order to exclude the influence of GMV on CBF, gender, years of education, and age were used as covariates, and the whole brain gray matter template was used as a mask. An independent sample *t*-test was performed to compare the difference in CBF between the observation group and the control group.

## 3. Results and Discussion

### 3.1. Performance Analysis of the Algorithm

**Figure 1:**
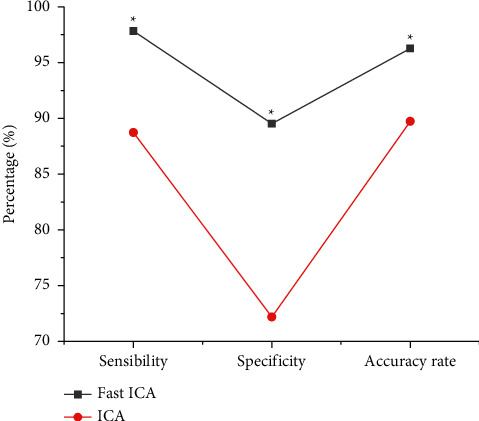
Performance comparison of the two algorithms.

In this research topic, the improved Fast ICA algorithm and the traditional ICA algorithm were compared and analyzed. The sensitivity, specificity, and accuracy of the algorithm are shown in Figure 1. The sensitivity, specificity, and accuracy of the Fast ICA algorithm were 97.83%, 89.52%, and 96.27%, respectively; the sensitivity, specificity, and accuracy of the traditional ICA algorithm were 88.73%, 72.19%, and 89.72%, respectively. There were obvious differences between the two groups, and the two were statistically remarkable (*P* < 0.05).

**Figure 2:**
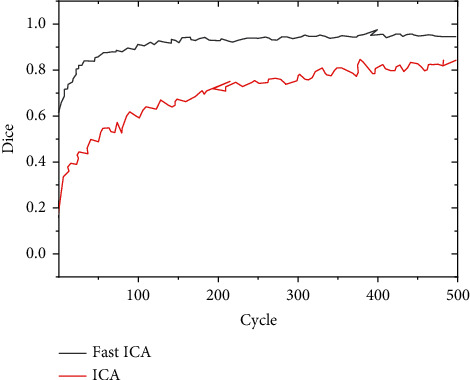
Comparison of Dice coefficients of different algorithms under different cycle periods.

**Figure 3:**
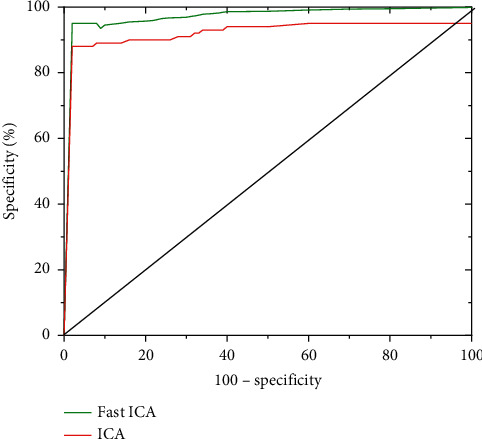
Comparison of the AUC values of the two algorithms.

In different training cycles, the Dice coefficients of the MRI images processed by the Fast ICA algorithm in this study were compared with those processed by the traditional ICA algorithm, and the results are presented in Figure 2. With the increase of training cycle, the Dice coefficients of different algorithms all showed an obvious upward trend. The Dice coefficients of Fast ICA algorithm in this study were greater than the coefficients of the traditional ICA algorithm in different training cycles; the Dice coefficient of Fast ICA algorithm reaches 0.967 at most, while the Dice coefficient of the traditional ICA algorithm was 0.738 at most.  Figure 3 reveals that the area under the curve (AUC) of the Fast ICA algorithm was 0.978, and the AUC of the traditional ICA algorithm was 0.773. Therefore, the Fast ICA algorithm was better greatly than the traditional ICA algorithm (*P* < 0.05).

### 3.2. Comparison of General Data of Patients from the Two Groups

**Figure 4:**
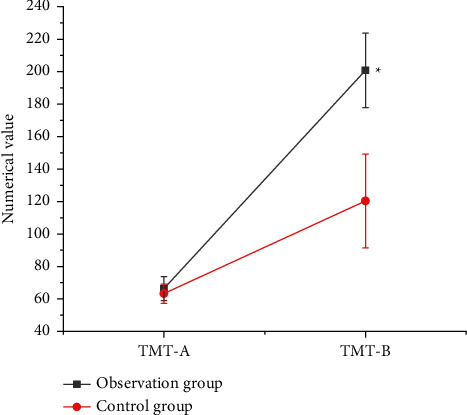
Comparison on TMT test results of patients from the two groups.  ^*∗*^*P* < 0.05 compared with the control group.

As shown in Figure 4, the connection TMT-A of the observation group was 66.37 ± 7.39, and that of the control group was 63.28 ± 5.92; the connection TMT-B of the observation group and the control group was 200.83 ± 22.93 and 120.35 ± 28.93 in turn. It was found that there were statistically huge differences in TMT-B between the two groups (*P* < 0.05).

### 3.3. Analysis of the Results of the Patients' Brain Function Area

**Figure 5:**
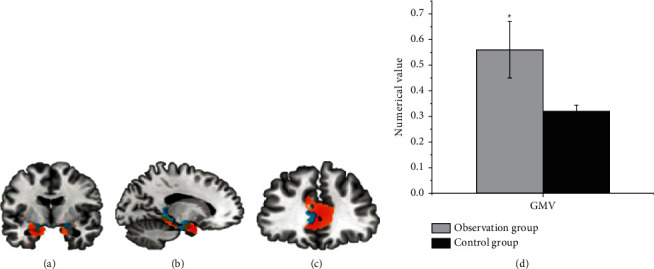
The infarct focus located in the basal ganglia area (a); the lateral ventricle (b); the thalamus position (c); and GMV comparison between the two groups (d) (note: ^*∗*^meant that the GMV in the SMA area of the contralateral hemisphere in patients with subcortical cerebral infarction and good recovery of motor function in the observation group increased hugely compared with the control group (*P* < 0.05)).

Figure 5 shows that the GMV of the auxiliary motor area (SMA) of the contralateral cerebral hemisphere in the observation group was 0.56 ± 0.11 and that of the control group was 0.32 ± 0.023. The gray matter area of the observation group was significantly increased compared with the control group, and no area of GMV reduction was found.

### 3.4. Analysis of CBF Results

**Figure 6:**
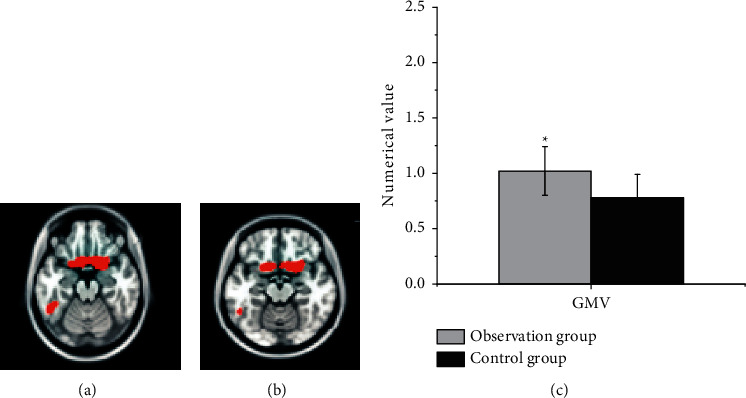
Comparison of CBF in the SFG of the healthy hemisphere in patients with good motor function recovery from the observation group. (a) The fMRI image of the patient before treatment, (b) The fMRI image of the patient after treatment. (c) The comparison on CBF between the two groups.  ^*∗*^*P* < 0.05 compared with the control group.

The comparison results of CBF in the two groups of patients showed that the CBF of the healthy superior frontal gyrus (SFG) and the healthy superior marginal gyrus (SMG) of the observation group with good recovery of motor function were 1.02 ± 0.22 and 1.53 ± 0.61 in sequence. What is more, both showed an increasing trend (Figure 6(c)), which was statistically different from the control group (*P* < 0.05), and there was no brain area with reduced CBF (Figures 6(a) and 6(b)). The brain areas with increased CBF in the healthy SFG and healthy SMG were used as ROI1 and ROI2 in turn for analysis of resting state functional connectivity.

**Figure 7:**
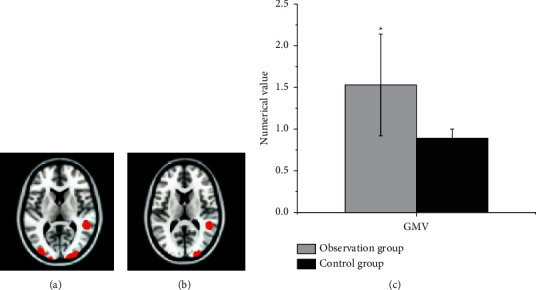
Comparison of CBF in the SMG of the healthy side of the observation group with good motor function recovery. (a) The fMRI image of the patient before treatment. (b) The fMRI image of the patient after treatment. (c) The comparison on CBF between the two groups.  ^*∗*^*P* < 0.05 compared with the control group.

After GMV correction, the CBF of the healthy SFG and healthy SMG of the subcortical cerebral infarction patients with good motor function recovery in the observation group increased significantly in contrast to the CBF of the control group (Figure 7). These results were consistent with the increase in CBF before GMV correction.

### 3.5. rsFC Result Analysis

**Figure 8:**
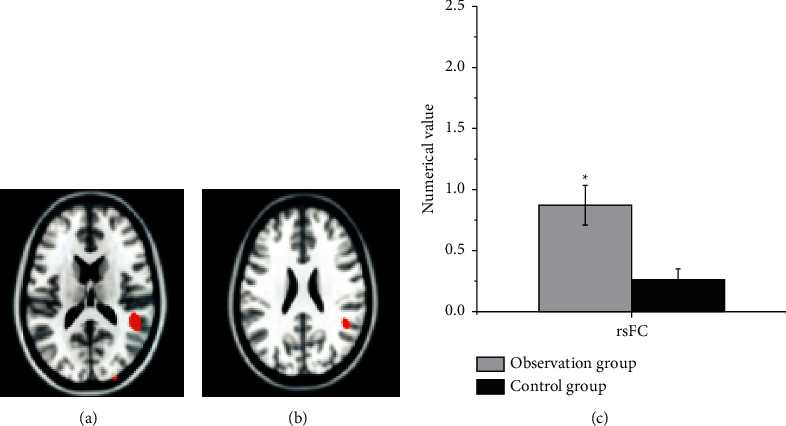
Analysis results of resting state functional connection between the two groups of patients. (a) The fMRI image of the patient before treatment. (b) The fMRI image of the patient after treatment. (c) The comparison on rsFC between the two groups.  ^*∗*^*P* < 0.05 compared with the control group.

The functional connection ROI analysis of the whole brain was performed on the healthy SFG and healthy SMG. The results are shown in Figure 8, suggesting that the resting-state functional connectivity (rsFC) values of patients from the observation group and the control group were 0.872 ± 0.163 and 0.261 ± 0.09, respectively. Thus, there was a statistical difference between the two groups of patients (*P* < 0.05).

### 3.6. Comparison of the Results of Correlation Analysis between Patient Behavior Data and Image Data

**Figure 9:**
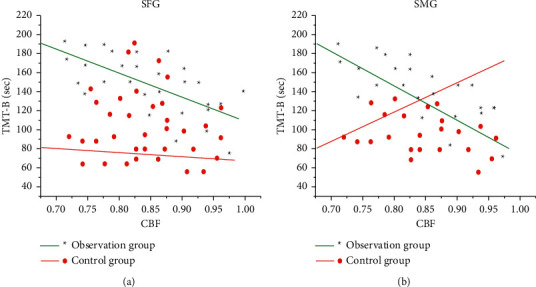
Correlation analysis between the CBF values and the connection TMT-B. (a) Correlation analysis on the CBF and the connection TMT-B in the healthy SFG. (b) Correlation analysis on the CBF and the connection TMT-B in the healthy SMG.

When the clinical basic indicators of the patients were consistent, the CBF values of the healthy SFG of the patients with good motor function recovery in subcortical cerebral infarction were *r* = −0.362 and *P*=0.0357 (Figure 9(a)), and the CBF values of the healthy SMG were *r* = −0.369 and *P*=0.0421 (Figure 9(b)). Thus, the CBF values of the healthy SFG and healthy SMG were negatively correlated with the results of connection TMT-B. However, there was no correlation between the cognitive function of GMV and rsFC in patients with cerebral infarction (*P* > 0.05).

## 4. Discussion

Cerebral infarction is caused by stenosis and occlusion of cerebral blood vessels due to various reasons, resulting in insufficient blood supply to the brain to cause hypoxia and brain tissue necrosis. The clinical symptoms are manifested as motor, sensory, cognitive, and other dysfunctions. The disease has a high incidence, and the course of the disease is longer, which has a great impact on the work and life of the patient. fMRI technology has developed rapidly in recent years, which can explore the pathological and physiological changes of cerebral infarction from multiple perspectives and provide sufficient imaging basis for clinical diagnosis. In this study, the improved FAST ICA algorithm was applied to optimize the image processing of fMRI so as to improve the diagnostic accuracy of fMRI imaging for cerebral infarction patients. The results showed that the sensitivity, specificity, and accuracy of the Fast ICA algorithm in the experimental group were 97.83%, 89.52%, and 96.27%, respectively, which were greatly better than those of the control group, showing statistically significant differences (*P* < 0.05). The maximum Dice coefficient of Fast ICA algorithm was 0.967, while the maximum Dice coefficient of traditional ICA algorithm was 0.738. The AUC values of FAST ICA algorithm and traditional ICA algorithm were 0.978 and 0.773 in turn, so the FAST ICA algorithm was obviously better than traditional ICA algorithm (*P* < 0.05). The research results were similar to the research findings of Zan et al. [22], and both showed that the diagnostic accuracy and other performance of the optimized Fast ICA algorithm were significantly improved.

In the diagnosis of patients with cerebral infarction, the GMV of the contralateral cerebral hemispheric SMA in the observation group and the control group was 0.56 ± 0.11 and 0.32 ± 0.023 in sequence. Compared with the control group, the gray matter area was significantly increased, and no area with a decreased GMV was found, which indicated that the brain structure of the contralateral cortex underwent plastic changes. SMA is an auxiliary motor area of the brain and an important part of the brain motor network. Houkin et al. [15] pointed out that the patient's motor function gradually recovered and the gray matter volume in the healthy SMA area increased compensatory, with the extension of time, suggesting that the brain structure had obvious plasticity. Compared with the normal control group, the CBF of the observation group with good motor function recovery in the healthy SFG (1.02 ± 0.22) and healthy SMG (1.53 ± 0.61) displayed an increasing trend. Thus, there was a statistically significant difference between the two groups (*P* < 0.05). The rsFC values of functional connectivity in resting state from the observation group and the control group were 0.872 ± 0.163 and 0.261 ± 0.09, respectively, and there was a statistically huge difference between the two groups (*P* < 0.05). *r* = −0.362 and *P*=0.0357 in the CBF value of the healthy SFG of the patients with good motor function recovery in subcortical cerebral infarction, and CBF value of the healthy SMG was *r* = −0.369 and *P*=0.0421. Besides, the CBF value of the healthy SFG and the healthy SMG was negatively correlated with the results of the connection TMT-B. The research of Zhang and Shen [23] found that there was a marked correlation between the blood flow in the left corner and the posterior part of the left middle temporal gyrus in patients with subacute cerebral infarction, as well as dyslexia. Nah et al. [16] applied ASL to longitudinal investigate the relationship between CBF and motor function recovery in patients with cerebral infarction, finding that CBF in the SMA on the healthy side of patients with better motor function recovery showed a significant downward trend, and the CBF between the two hemispheres was gradually balanced. Patients with poor motor function recovery would have continuous low perfusion state in the motor sensory cortex. It also meant that the imbalance of blood perfusion ratio in the sensorimotor cortex was likely to cause poor motor function recovery after cerebral infarction. The correlation analysis of this study found that there was a negative correlation between CBF values of healthy SMG and healthy SFG and the connection TMT-B. Furthermore, connection TMT-B reflected the ability to execute; the shorter the time of connection TMT-B, the stronger the ability to execute. In other words, the CBF values of healthy SMG and healthy SFG could enhance the leadership of patients. The results of this study were consistent with previous studies, showing that CBF in brain areas was associated with motor or cognitive function.

## 5. Conclusion

In this study, 50 patients with cerebral infarction were selected as the observation group, and 30 healthy people were selected as the control group. The patients in observation group were examined with fMRI based on Fast ICA algorithm, and the patients in control group were examined with fMRI based on conventional ICA algorithm. The results revealed that fMRI based on Fast ICA algorithm showed a good diagnostic effect in detecting brain functional area changes in patients with cerebral infarction. Patients with cerebral infarction with good motor function recovery had changes in brain structure and functional plasticity, and the plastic reconstruction of brain functional areas was conducive to the recovery of patients' motor and cognitive functions. The disadvantage was that the sample size of the selected patients was small and the source was single, which may have some influence on the results. It would increase the sample size of patients and conduct multicenter and large sample size analysis in future. In short, the results of this article provided a reliable reference for the examination and diagnosis of brain functional areas in patients with cerebral infarction.

## Data Availability

The data used to support the findings of this study are available from the corresponding author upon request.
